# Psychometricre-evaluation of the locus of control short scale (IE-4) in the German general population: evidence of acquiescence bias

**DOI:** 10.1186/s40359-026-05101-4

**Published:** 2026-07-04

**Authors:** Katja Petrowski, Julian Duhm, Vanessa Renner, Bjarne Schmalbach, Bernhard Strauß, Elmar Brähler

**Affiliations:** 1 Medical Psychology and Medical Sociology, Medical Center of the Johannes Gutenberg University Mainz, Duesbergweg 6, Mainz, 55128 Germany; 2 Clinical Psychology, Hochschule Fresenius Wiesbaden, Wiesbaden, Germany; 3 Institute of Psychosocial Medicine, Psychotherapy and Psychooncology, Jena University Hospital, Friedrich Schiller-University Jena, Jena, Germany; 4 Department of Psychosomatic Medicine and Psychotherapy, Medical Center of the Johannes Gutenberg University Mainz, Mainz, Germany; 5 Clinic and Polyclinic for Psychosomatic Medicine and Psychotherapy, Rostock University Medical Center, Rostock, Germany

**Keywords:** Locus of control, Acquiescence bias, Confirmatory factor analysis, Random intercept

## Abstract

The Locus of Control short scale (IE-4) is a screening instrument for locus of control (LOC). Assessment of LOC is highly relevant within psychological research and social sciences. The aim of the current study was to evaluate the screening tool in a large representative sample, with special focus on its dimensionality and the presence of acquiescence bias. The current sample (*N* = 2522; Age: M = 48.50 years (*SD* = 17.75); 50.1% female) was representative for the German adult population with respect to gender, age, and education. After conducting a CFA including an uncorrelated method factor, model fit indices generally indicated a good fit. Additionally, we found evidence of good reliability. Results from this study suggest that the IE-4 is best considered as a unifactorial measure when accounting for acquiescence bias.

Locus of Control emerged as part of the social learning theory and represents an individual difference variable on generalized perceived control over life events. In theory, LOC is shaped by learning experience acquired in specific social settings, assuming that an individual’s learning experience from the past directly impacts that person’s perception and behavior in similar social situations with respect to perceived control over life events [15]. LOC is a stable aspect of personality that provides a generalizing view on self-perception and the environment, thereby representing a key component in understanding and predicting human behavior [6, 21]. While internal reinforcement represents an individual’s belief that life events are under his or her control, external reinforcement is defined as a perceived lack of control over life events. Externals thus believe that things in life depend on fate, chance, or powerful others [16]. LOC has been shown to significantly impact and have moderating effects on a number of highly relevant variables including both physical and mental health [1, 4, 10, 18].

Over the decades, different approaches have been taken towards the scientific assessment of reinforcement. Largely based off the work of Albert Bandura, Rotter [15] introduced the Internal/External Locus of Control Scale (ROT-IE). However, despite thorough validation as for use over time and across different samples and cultures [7, 13, 22, 23], Levenson [8, 9] questions the unidimensional approach of the ROT-IE and instead proposes a multidimensional concept of LOC. In fact, the Internal, Powerful Others, and Chance-Scale (IPC) offers a tripartite, multidimensional view on LOC. Krampen [6] as well as Jakoby & Jacob [2] built on and extended this multifactorial approach, which was later empirically tested and served as a framework for the IE-4 scale [5]. The recent study by Nießen and colleagues [13] in particular deserves to be highlighted here for its scope and rigor in validating the IE-4 – both, in terms of its ostensible two-factorial structure as well as external criteria.

## Unclear factor structure and potential methodological remedy

As shown above, previous research has had proponents of both a unifactorial and a multifactorial conception of LOC. And yet up until this point the issue of methodologically-founded pseudo-factors is completely unstudied with regard to the the LOC [20], specifically acquiescence. In questionnaires, there is a clear tendency for respondents to generally reply in accordance with a proposed statement. This effect is independent of the actual content of the presented items and can also vary in strength across individuals. Especially in the presence of reverse-coded items, this effect becomes problematic from a statistical point of view because it implies an additional pseudo-content factor. Schmalbach et al. [20] have shown that modeling the acquiescence bias as defined by Maydeu-Olivares and Coffman [11] in their Random Intercept Model (RIM) can clear up many factorial ambiguities in favor of more theoretically parsimonious models. The RIM is defined as a confirmatory factor analysis model that includes a method factor that is unit loading onto all items. In addition, it is constrained to be uncorrelated to the content factor of the model. Thus, the random intercept factor in effect captures all variance that has a uniform effect on all items of a scale – irrespective of the polarity of coding. It should be emphasized here that this method is only sensible with scales that feature reverse-coded items.

### The present study

The goal of the present study is clarifying the dimensionality of the IE-4, since no previous study has considered the presence of method effects, namely that of acquiescence bias. To this end, we will compare a unifactorial (1FM), two-factorial (2FM), and a unifactorial model with a random intercept – representing (1FRIM) uniform acquiescence bias [11]. In line with previous research, we expect the 1FM to perform badly. Further, we expect the 2FM and the 1FRIM to be roughly equivalent in terms of fit. This should be considered evidence for the 1FRIM’s preferability because of its theoretical parsimony. If instead the 2FM exhibts significantly better fit, this should be considered evidence for the existence of two substantive factors which cannot be explained by acquiescence bias.

## Methods

### Sample and procedure

The present sample was recruited with the assistance of the demographic consulting company USUMA (Berlin, Germany). Participant selection was based on electoral districts, a random-route procedure, and the Kish selection grid. Only individuals with sufficient command of the German language were included in this study. Given a margin of error of 2% and a confidence level of 95% a sample representative of the German general population would require at least 2,401 respondents. Thus our sample of *n* = 2,522 can be considered representative of the German general population.

Participants were visited at home by a trained interviewer, and after giving informed consent, they filled in several self-report questionnaires. Participants were also informed of general study procedures, data collection, and anonymization of all personal data. Additionally, a detailed data privacy statement was delivered by the interviewer. Overall, the study adhered to the guidelines of the ICMJE Recommendations for the Protection of Research Participants and the Helsinki Declaration as revised 2008, and it was approved by the ethics committee of the Medical faculty of the University of Leipzig (355/22-ek).

### Instruments

The current study used the German version of the IE-4 [3, 5], which is a four-item measure that assesses internal and external locus of control using four 5-point Likert-scale items (see Table 1). Response options range from 1 (doesn’t apply at all) to 5 (applies completely).

**Table 1:** Loadings for the two competing models of the IE-4

	2FM		1FRIM	
Item			Content Factor	Method Factor
If I work hard, I will succeed.	.867		.747	.406
I‘m my own boss.	.761		.674	.385
Whether at work or in my private life: What I do is mainly determined by others.		.744	-.640	.326
Fate often gets in the way of my plans.		.655	-.591	.334

### Transparency and openness

All data, analysis code, and research materials are available upon request from the first author. Data analyses were carried out using R and the package *lavaan* [17]. Neither the study design nor the analysis plan were pre-registered. We report how we determined our sample size, all data exclusions (if any), all manipulations, and all measures in the study. The sample size was determined with the goal of representativeness (rather than statistical power). Specifically, we aimed for a sample size of at least 2,401 based on a population of 83 million, a margin of error of 2%, and confidence level of 95%.

### Statistical analyses

We fit confirmatory factor analysis models using full-information robust maximum likelihood estimation. To evaluate model fit, we examined fit indices and compared them to the customary cutoff values as reported by Schermelleh-Engel et al. [19]: Comparative Fit Index (CFI) and Tucker-Lewis Index (TLI) greater than .95 (.97) for acceptable (good) fit, as well as Root Mean Square Error of Approximation (RMSEA) and Standardized Root Mean Square Residual (SRMR) smaller than .08/.10 (.05) for acceptable (good) fit.

## Results

### Sociodemographic characteristics of participants

The initial sample size was N = 2522. There were a total of 49 (0.5%) missing values on the IE-4 items. Sociodemographic details of the sample are reported in Table 2.

**Table 2:** Sociodemographic characteristics of the sample

	*n*	%
Total	2522	100
Sex		
Male	1254	49.7
Female	1264	50.1
Diverse	4	0.2
Age, in years (Range 16–96)		
≤24	209	8.3
25–34	408	16.2
35–44	451	17.9
45–54	417	16.5
54–64	489	19.4
65–74	341	13.5
>74	207	8.2
Family		
Married	1165	46.2
Separated	41	1.6
Unmarried	736	29.2
Divorced	363	14.4
Widowed	215	8.5
Work		
Full-time	1224	48.7
Part-time	365	14.5
Unemployed	138	5.5
Retired	656	26.0
In training	139	5.5
Education		
<10 years	680	27.0
10 years	1178	46.7
>10 years	622	24.7
Student	42	1.7

Initially, we fit a 1FM, but expectedly this evinced unacceptable fit, χ^2^(2) = 322.73, p < .001, CFI = .834, TLI = .501, RMSEA = .291, SRMR = .084, a wide range of factor loadings from .409 to .831, and acceptable reliablility, ω = .721. We then tested two alternative models. Firstly, we tested the 2FM, splitting up the internal and external LOC items into two distinct latent variables. This model showed good fit, χ^2^(1) = 10.58, p < .001, CFI = .995, TLI = .972, RMSEA = .069, SRMR = .009. In addition, factor loadings now ranged between .655 and .867. Reliability for Internal Locus was good, ω = .798, but External Locus had unacceptable reliability, ω = .658.

Next, we fit the 1FRIM which showed almost identically good fit to the two-factorial model, χ^2^(1) = 10.62, p < .001, CFI = .995, TLI = .971, RMSEA = .070, SRMR = .008. The factor loadings on the content factor were between .591 and .747, while the loadings on the method factor were between .334 and .406. The resultant internal consistency estimates indicated that a majority of response variance can be attributed to the construct of interest, ω(Total) = .649, ω(method factor partialed out) = .806.

## Discussion

The aim of the current study was to further investigate the potential of the IE-4 as a cost and time effective tool for LOC assessment based on a representative sample of the general adult German population. Specifically, we focused on the question of its dimensionality. This contribution is the first to consider the impact of acquiescence bias for the IE-4.

As expected, model fit for the 1FM was poor whilst it was good for the 2FM, seemingly supporting that the two subscales of the IE-4 (internality vs. externality) in fact display two distinct dimensions. This lines up well with previous research such as the extensive validation by Nießen and colleagues [13]. However, model fit was almost identical for the 1FRIM. Due to its theoretical parsimony, we thus decided in favor of the 1FRIM. Our initial suspicion that in the case of the IE-4 acquiescence led to the emergence of a second factor can thus be regarded as confirmed. This is a common issue when reverse-keyed items are concerned, which usually leads to a negative impact on reliability and model fit by way of the acquiescence bias [11, 20]. In line with this, recent research suggests that reverse-keyed items are especially problematic with short inventories by impacting factorial structure, validity, and reliability [12].

Despite the considerable number of potential threats to factorial structure, results from the present study indicated acceptable psychometric properties for the IE-4 with respect to validity and reliability. This further underpins the utility of the scale as a brief assessment tool for LOC. Specifically, we found good internal consistency (ω = .806). This finding is in contrast to the mixed results obtained by Kovaleva et al. [5]. This again emphasizes the importance of explicitly modeling acquiescence effects, as they will strongly deteriorate reliability if left unaccounted for.

### Limitations and future reseach

In this study, we focused primarily on acquiescence. Past research has identified various types of response biases that could further add to our understanding of the concept of LOC and its measurement [14]. This includes phenomena such as item characteristic effects as well as item and measurement context effects. Thus, although only accounting for acquiescence proved sufficient to demonstrate the IE-4’s unidimensional nature, different biases should also be considered by future research.

#### Constraints on generality

The sample under consideration is explicitly limited to the German general population. It can be considered representative for this population based on the methodology and inspection of the distributions of the sociodemographic variables. However, the results should be replicated by future research in other cultures and demographics outside of Germany.

## Conclusion

In summary, our results align well with previous findings [5, 13]. We add to the understanding of LOC by clarifying the role of acquiescence in the observed response behavior in the presence of reverse-coded items. We conclude that the IE-4 is a highly suitable tool for economic assessment of LOC in adults and strongly recommend a unifactorial interpretation – with an emphasis on proper modeling of response biases.

## Data Availability

The data used in the analyses is available from the authors upon reasonable request.
